# Effects of life history and individual development on community dynamics: A review of counterintuitive consequences

**DOI:** 10.1111/1440-1703.12174

**Published:** 2020-10-08

**Authors:** André M. de Roos

**Affiliations:** ^1^ Institute for Biodiversity and Ecosystem Dynamics University of Amsterdam Amsterdam Netherlands; ^2^ The Santa Fe Institute Santa Fe New Mexico USA

**Keywords:** community structure, juvenile–adult asymmetry, population dynamics, population stage‐structure, structured population models

## Abstract

Even though individual life history is the focus of much ecological research, its importance for the dynamics and structure of ecological communities is unclear, or is it a topic of much ongoing research. In this paper I highlight the key life history traits that may lead to effects of life history or ontogeny on ecological communities. I show that asymmetries in the extent of food limitation between individuals in different life stage can give rise to an increase in efficiency with which resources are used for population growth when conditions change. This change in efficiency may result in a positive relationship between stage‐specific density and mortality. The positive relationship between density and mortality in turn leads to predictions about community structure that are not only diametrically opposite to the expectations based on theory that ignores population structure but are also intuitively hard to accept. I provide a few examples that illustrate how taking into account intraspecific differences due to ontogeny radically changes the theoretical expectations regarding the possible outcomes of community dynamics. As the most compelling example I show how a so‐called double‐handicapped looser, that is, a consumer species that is both competitively inferior in the absence of predators and experiences higher mortality when predators are present, can nonetheless oust its opponent that it competes with for the same resource and is exposed to the same predator.

## Graphical abstract

Individual life history is often ignored in dynamic models of interacting populations, which tend to be formulated in terms of total densities of identical individuals. However, the consequences of differences between conspecific individuals that arise from differences in their developmental state are substantial and often counterintuitive. This paper reviews these community consequences and discusses under what conditions life history differences will be important.

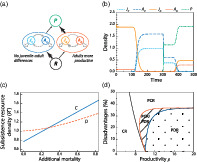

## 1 INTRODUCTION

Ever since Darwin's “On the Origin of Species” differences between individuals of the same species have been at the forefront of ecological and evolutionary thought. Variation among conspecific individuals in heritable traits provides the raw material on which selection can act and is therefore naturally at the core of evolutionary biology. In ecology differences between conspecifics are considered equally important (Bolnick et al., 2003, 2011; Moran, Hartig, & Bell, 2016), but the situation is more complicated as individuals can differ in non‐heritable traits as well. Within populations individuals can for example differ in competitive ability (Duffy, 2002), anti‐predator defense (Becks, Ellner, Jones, & Hairston, 2010), resource use (Andersson, Byström, Persson, & de Roos, 2005; Bolnick et al., 2003; Snorrason et al., 1994), or personality (Wolf & Weissing, 2012), which may be the result of phenotypic plasticity across varying environmental conditions instead of heritable differences between individuals. In an important review Bolnick et al. (2011) called for more attention of the effect of intraspecific variation on ecological dynamics, while pointing out that “…*ecological theory typically focuses on predicting the dynamics of species’ abundances over time without regard to particular phenotypes. Consequently, many models of species' interactions implicitly assume that all conspecific individuals are effectively interchangeable*.” In recent years the importance of intraspecific variation in ecological processes has been recognized to an increasing extent, in particular because of the focus on trait‐based ecology (Moran et al., 2016; Palkovacs & Post, 2008; Werner & Peacor, 2003).

Given the importance attributed to individual differences it is remarkable that differences between individuals originating from their developmental state draw much less attention than differences due to heritable traits and phenotypic plasticity. Life history is the most fundamental feature that sets individual organisms apart from elementary particles in physics or molecules in chemistry. Individuals in different stages of their life history contribute to ecological dynamics in fundamentally different ways, most importantly because only adults reproduce, whereas juveniles grow, develop and mature. Even though Bolnick et al. (2011) acknowledge that differences in developmental state are ecologically significant (Polis, 1984), the research agenda they subsequently propose ignored such life history related differences and focused on densities of different phenotypes that have their own population dynamics. Differences in age, stage, or body size between individuals in the same population are taken into account when considering the demography and dynamics of single populations (Caswell, 2001; Ellner, Childs, & Rees, 2016), but models of interacting populations or ecological communities almost purposefully neglect them, calling on an argument that it would reduce generality of the modeling results.

Studies of eco‐evolutionary dynamics provide an example of this bias to ignore differences related to life history in population dynamics. These studies have established the occurrence of consumer–resource cycles in which consumers and resources fluctuate in antiphase (i.e., with a lag between maxima of the two populations equal to half the cycle period) as the hallmark of rapid evolution in the trade‐off between predator defence and competitive ability traits (Fussmann et al., 2005; Hiltunen, Hairston, Hooker, Jones, & Ellner, 2014; Scheuerl, Cairns, Becks, & Hiltunen, 2019). Yet, it has long been known that such antiphase consumer–resource cycles also arise as a consequence of asymmetric competition between juvenile and adult consumers (de Roos, Metz, Evers, & Leipoldt, 1990; de Roos & Persson, 2003; Metz, de Roos, & van den Bosch, 1988). Despite that the latter mechanism provides a more straightforward and hence more parsimonious explanation for the antiphase consumer–resource cycles, the interest in such individual life history processes as explanations for population and community phenomena pales in comparison with the search for explanations in terms of eco‐evolutionary dynamics and effects of rapid evolution on ecology (Hendry, 2016, 2019; Palkovacs et al., 2009; Pelletier, Garant, & Hendry, 2009).

Ecological differences related to different life history stages were the subject of a number of studies in the 1980s (Ebenman & Persson, 1988; Werner & Gilliam, 1984; Wilbur, 1988) and were considered by Schoener (1986) to be more important than other types of phenotypic variation, as he stated that “*for the most part, the important between‐phenotype variation in populations occurs between sex and age classes*” (Schoener, 1986, p. 119). Werner and Gilliam (1984) called attention to the widespread occurrence of ontogenetic niche and habitat shifts in the majority of species and discussed the possible consequences for community structure and dynamics. Body size was already at that time recognized as an important individual attribute, determining its energy requirements, its potential to exploit different resources and its vulnerability to predators. Size‐ and stage‐dependent trophic relations were shown to result in strong competition among juvenile predators, retarding their maturation and causing a lack of response in adult predator densities to increased levels of their resource (Neill, 1988; Neill & Peacock, 1980). Such “juvenile bottlenecks” were considered especially important for the dynamics of many fish communities as individual predators were generally growing through the same range of body sizes as their future prey (Mittelbach, 1983; Persson, 1985, 1988; Werner, 1986). It was shown that often the critical feature of interactions between species with distinct body sizes is indeed not how adults interact, but how the larger species is able to recruit through juvenile stages that have body sizes (and hence size‐dependent niche properties) comparable with the smaller species. For example, Neill (1975) demonstrated experimentally that a small zooplankter, *Ceriodaphnia*, could monopolize the food sizes used by juveniles of much larger species and thereby outcompete and actually drive to extinction a large zooplankter like *Daphnia magna*. Adult *Daphnia* introduced into the system survived and produced many offspring, of which not a single one survived the competition by *Ceriodaphnia* (see Persson, de Roos, & Byström, 2007 for a similar example involving fish). Despite this interest in stage‐specific interactions Werner and Gilliam (1984) pointed out that also at that time ecologists were paradoxically paying in general less attention to the community consequences of the 10‐fold variation in body size that is regularly observed within species than to the difference in body size by a factor of 2 that was considered necessary for coexistence of competing species (Schoener, 1974).

Mathematically rigorous theory development about stage‐dependent interactions was initially limited to the individual level, giving rise to concepts like the “μ/g” rule (Gilliam, 1982; Werner & Gilliam, 1984) for the optimal timing of the switch between two different ontogenetic niches or habitats. Stage‐specific interactions were furthermore shown to result in population cycles that were different from the classical cycles originating from predator–prey interactions (Gurney & Nisbet, 1985; Murdoch, Briggs, & Nisbet, 2003; Nisbet & Gurney, 1983). In an illuminating study Gurney and Nisbet (1985) showed how competition for resources among insect larvae could give rise to cycles in insect populations with a periodicity equal to 1–2 times or 2–4 times the juvenile delay, depending on whether larvae would immediately experience a negative impact from the resource competition or only after they had matured to the adult stage. More specifically, if increased larval competition translated into a density dependent increase in larval mortality or age at maturation population cycles resulted with a periodicity equal to 1–2 times the juvenile period. If, on the other hand, increased larval competition for resources translated later in life in a density dependent decrease in pupation success or adult fecundity, population cycles emerged with a periodicity equal to 2–4 times the juvenile delay. In both cases however the population was dominated by a single cohort of individuals born within a short time span of each other, such that these cycles were referred to as single‐generation cycles. These single‐generation cycles were in later studies shown to occur more generally as a result of asymmetric competition between juveniles and adults for a shared resource (de Roos, Metz, & Persson, 2013; de Roos & Persson, 2003, 2013; Persson & de Roos, 2013; Persson, Leonardsson, de Roos, Gyllenberg, & Christensen, 1998).

Mathematically rigorous theory about the effect of stage‐ and size‐dependent interactions on community structure has only been developed in the last two decades (de Roos & Persson, 2002, 2013; Miller & Rudolf, 2011; Nakazawa, 2010, 2011; Schreiber & Rudolf, 2008). These studies focused in particular on how asymmetric competition between juveniles and adults, size‐dependent predation and ontogenetic niche shifts lead to different types of community steady states and to what extent these stage‐dependent interactions led to different predictions regarding community structure than expected on the basis of classical theory in terms of unstructured populations. In this paper I provide a short review of the highlights that have emerged from these theoretical developments regarding stage‐specific interactions and community structure.

## 2 DENSITY OVERCOMPENSATION THROUGH MORE EFFICIENT RESOURCE USE: INCREASING ABUNDANCES DESPITE INCREASES IN MORTALITY

In consumer–resource systems with juvenile and adult consumers competing for a shared resource, changes in population structure with changing conditions occur when either juveniles or adults are limited more by food availability (de Roos, 2018; de Roos et al., 2007; de Roos & Persson, 2013). In particular, when adult reproduction is more limited by food availability than juvenile maturation, reproduction constitutes a bottleneck in the consumer's life history at low mortality. An increase in mortality relaxes this bottleneck and hence leads to an increase in the fraction of juveniles in the population. Similarly, when maturation is more limited by food than reproduction, the fraction of juveniles will decrease with increasing mortality as the latter will relax the juvenile maturation bottleneck. The increase or decrease in the fraction of juveniles can be so large that it overrides the decrease in overall population density such that as a consequence either juvenile or adult abundance increases rather than decreases with the increasing mortality.

The necessary conditions for this overcompensation in stage‐specific density in response to increasing mortality to occur can be worked out from the following simple model for a population of juvenile and adult consumers foraging on a shared resource (de Roos, 2018):

(1) $$\begin{array}{l} \frac{\mathrm{dR}}{\mathrm{dt}}=p\left(R\right)-f_{J}\left(R\right)J-f_{A}\left(R\right)A\\ \frac{\mathrm{dJ}}{\mathrm{dt}}=g_{A}\left(R\right)A-g_{J}\left(R\right)J-\mu_{J}J\\ \frac{\mathrm{dA}}{\mathrm{dt}}=g_{J}\left(R\right)J-\mu_{A}A. \end{array}$$

In these model equations the variables *R*, *J* and *A* refer to the densities of resource, juvenile and adult consumers, respectively. Resource density increases through resource replenishment described by the function *p*(*R*), whereas it decreases through foraging by juvenile and adult consumers at per capita rates equal to *f*_*J*_(*R*) and *f*_*A*_(*R*), respectively. Adult consumers have a per capita fecundity equal to *g*_*A*_(*R*), whereas juvenile consumers mature at a per capita rate *g*_*J*_(*R*), while juvenile and adult consumers experience per capita mortalities equal to *μ*_*J*_ and *μ*_*A*_, respectively. As a very natural assumption the functions *f*_*J*_(*R*), *f*_*A*_(*R*), *g*_*J*_(*R*) and *g*_*A*_(*R*) all increase with an increase in resource density, reflecting that ingestion, maturation and reproduction are higher at higher food availability. Without making any further assumptions the response of the consumer densities due to an increase in either juvenile or adult mortality can be assessed (de Roos, 2018). Such an increase in mortality, whether it is of juveniles or of adults, will necessarily increase the resource density in the consumer–resource equilibrium of the system as consumers will have to mature faster and reproduce more to make up for the increase in mortality. Given that the functions *g*_*J*_(*R*) and *g*_*A*_(*R*) are increasing functions of resource density, more rapid maturation or higher fecundity can only be achieved at a higher equilibrium resource density.

When the increase in equilibrium resource density in response to an increase in juvenile or adult mortality translates into an increase in the resource productivity $p\left(\tilde{R}\right)$ at equilibrium total consumer abundance can increase with mortality. This requires that resource productivity *p*(*R*) is an increasing function of resource density and the derivative *p*^′^(*R*) is hence larger than 0, such as occurs when the resource follows a logistic growth function. The increase in total consumer abundance is actually independent of population structure and also occurs in unstructured consumer–resource models (Abrams, 2009; Abrams & Matsuda, 2005). Abrams and Matsuda (2005) were the first to describe this phenomenon and called it the “Hydra‐effect” (see Glossary). Notice that mechanistically in continuous‐time consumer–resource models the Hydra effect comes about because the overall productivity of the resource increases with the increase in equilibrium resource density which is required to compensate for the higher consumer mortality.

In contrast, when an increase in equilibrium resource density does not increase resource productivity (occurring when *p*^′^(*R*) ≤ 0), total consumer abundance will always decrease with an increase in juvenile or adult mortality. Analysis of the model equations (1) for this case shows that despite this decrease in total consumer abundance the stage‐specific density of either juvenile or adult consumers can nonetheless increase in equilibrium with an increase in mortality (de Roos, 2018). In particular, juvenile consumer density at equilibrium can increase with an increase in adult mortality if the following inequality holds at the equilibrium resource density $\tilde{R}$:

(2) $${\left(\frac{f_{A}\left(\tilde{R}\right)}{g_{A}\left(\tilde{R}\right)}\right)}^{\prime }<0,$$

whereas adult consumer density at equilibrium can increase with an increase in both juvenile and adult mortality if the inequality:

(3) $${\left(\frac{f_{J}\left(\tilde{R}\right)}{g_{J}\left(\tilde{R}\right)}\right)}^{\prime }<0,$$

holds at the equilibrium resource density $\tilde{R}$ (de Roos, 2018). In biological terms inequality (2) expresses that a small increase in equilibrium resource density, as result of an increase in mortality, will translate into a disproportionally large increase in adult fecundity $g_{A}\left(\tilde{R}\right)$ compared to the increase in adult food intake $f_{A}\left(\tilde{R}\right)$. Loosely speaking, the increase in equilibrium resource density results in a more efficient use by adults of the food they ingest as the ratio of their food intake rate and fecundity $f_{A}\left(\tilde{R}\right)/g_{A}\left(\tilde{R}\right)$ decreases at higher mortality. Similarly, inequality (3) expresses that with an increase in equilibrium resource density resulting from an increase in mortality juvenile consumers use the food they ingest more efficiently as the ratio of their food intake and maturation rate $f_{J}\left(\tilde{R}\right)/g_{J}\left(\tilde{R}\right)$ decreases.

Compared to the Hydra effect which comes about because of an increase in productivity with increasing equilibrium resource density, the stage‐specific overcompensation in juvenile or adult consumer density hence results from a more efficient conversion by consumers of the available resource productivity into processes like maturation and reproduction that contribute effectively to population growth (see Glossary). An obvious and natural condition that would allow such a more efficient use of resources to occur is when at low food availability maturation or reproduction would come to a complete halt because juvenile or adult consumers would be using all the food they ingest to cover their own metabolic maintenance costs for persistence. Energy demands to cover maintenance costs for individual subsistence are an essential feature of life that is not accounted for in basic ecological theory, as models generally assume that the reproduction rate of consumers is proportional to their intake of resources (Abrams & Ginzburg, 2000; Arditi & Ginzburg, 1989; May, 1972). Energy losses through maintenance, however, are much larger than losses through natural mortality (Yodzis & Innes, 1992). Estimation of lifetime energy expenditure for example indicate that in mammals and birds resting metabolism alone, not even including costs of activity, requires the equivalent of 100–1,000 times an individual's body weight (Speakman, 2005). Considered at the population level these estimates translate into losses through resting metabolism being 2 to 3 orders of magnitude larger than losses through mortality.

Given that metabolic maintenance costs for persistence are an inevitable fact of life and hence maturation of juveniles and reproduction by adults necessarily stop when food densities become too low, it can be postulated that the inequalities (2) and (3) will generally hold in any ecological system. However, the inequalities (2) and (3) represent only necessary and not sufficient conditions for stage‐specific densities to increase with mortality. For example, when juvenile and adult feeding rate, *f*_*J*_(*R*) and *f*_*A*_(*R*), as well as juvenile maturation rate and adult fecundity, *g*_*J*_(*R*) and *g*_*A*_(*R*), are proportional to each other stage‐specific increases in density with mortality do not occur, irrespective of whether or not inequalities (2) and (3) hold (de Roos, 2018). For stage‐specific densities to increase with increasing mortality some type of ontogenetic asymmetry (see Glossary) has to occur between juveniles and adults, such that one of these life history stages is at low mortality more limited by food availability than the other (de Roos et al., 2013; Persson & de Roos, 2013). It should also be noted that in the model equations (1) juvenile density cannot increase with an increase in juvenile mortality, whereas it can increase with increasing adult or stage‐independent mortality and adult density can increase with any type of mortality increasing (de Roos, 2018). In contrast, in size‐structured models that also account for juvenile growth in body size in addition to juvenile maturation, the biomass density of juvenile consumers can increase with an increase in juvenile mortality (de Roos et al., 2007; de Roos & Persson, 2013). In consumer–resource models that account for somatic growth in addition to maturation and reproduction the biomass density of juvenile and adult consumers at equilibrium can therefore increase with an increase in mortality irrespective of the stage‐specificity of the mortality, a phenomenon referred to as biomass overcompensation (see Glossary). This stage‐specificity will modify quantitatively the extent of the stage‐specific biomass increase, but not the qualitative response that juvenile biomass density in equilibrium will increase when adults are more limited by food availability and vice versa that adult biomass density will increase when juveniles are more limited by food availability.

**FIGURE 1:**
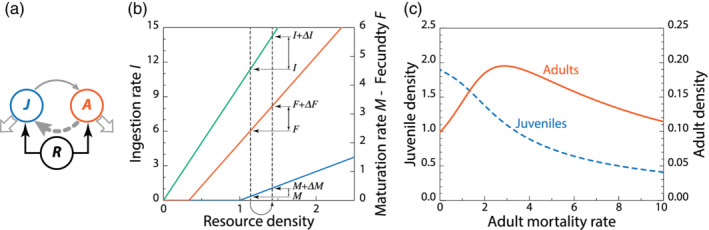
An increase in adult mortality increases adult density in a stage‐structured consumer–resource model with a juvenile bottleneck. (a) Juveniles and adults feed on a shared resource at equal rates but adults use ingested food more effectively such that juveniles experience a maturation bottleneck. (b) If maintenance requirements make that maturation (*M*) and reproduction (fecundity *F*) stop at low resource densities, the more effective use of resources by adult consumers entails that with an increase in resource density maturation rates will increase disproportionally more (Δ*M*/*M*) than fecundity (Δ*F*/*F*) and ingestion rates (Δ*I*/*I*). (c) Increases in adult mortality rate translate into increased resource densities that relax the juvenile maturation bottleneck and consequently lead to higher equilibrium densities of adult consumers. For model formulation and parameter values see main text. [Color figure can be viewed at wileyonlinelibrary.com]

Figure 1 shows a concrete example of the model system (1) in which juvenile and adult foraging rates are both assumed to equal *αR* (*f*_*J*_(*R*) = *f*_*A*_(*R*) = *αR*) with *α* = 10, while juvenile maturation and adult fecundity are assumed equal to *g*_*J*_(*R*) = max(*γR* − *T*, 0) and *g*_*A*_(*R*) = max(*βR* − *T*, 0), respectively, with *β* = 3, *γ* = 1 and *T* = 1. Resource productivity is assumed to be constant, *p*(*R*) = *ρ*, and equal to *ρ* = 20, while the background mortality of juvenile and adult consumers is assumed to equal *μ*_*J*_ = *μ*_*A*_ = 0.1 (see Table 1 for a list of all model parameters). The maximum functions in the juvenile maturation rate *g*_*J*_(*R*) and the adult fecundity *g*_*A*_(*R*) imply that maturation and reproduction do not occur at resource levels *R* below *T*/*γ* and *T*/*β*, respectively, in which the threshold parameter *T* phenomenologically represents the somatic maintenance costs of juvenile and adult consumers. Figure 1b illustrates that a small increase in food ingestion rate for both juveniles and adults translates into a much larger proportional increase in maturation rate than in adult fecundity, as a result of the parameter choice that *β* > *γ* and juveniles are hence more limited by food availability than adults. The increase in juvenile maturation rate is also disproportionally large compared to the increase in their food ingestion rate, such that the ratio *f*_*J*_(*R*)/*g*_*J*_(*R*) indeed decreases. As a consequence of the relaxation of the juvenile maturation bottleneck the fraction of adult consumers in the population increases more than the decrease in total consumer density, such that adult consumer density increases with an increase in adult mortality (Figure 1c).

**TABLE 1:** Model parameters with default values

Symbol	Default value	Interpretation
*ρ*	20	Productivity of the resource
*α*	10	Attack rate parameter of juvenile and adult consumer on resource
*T*	1	Maintenance costs parameter of juvenile and adult consumers
*γ*	1	Net resource assimilation rate parameter of juvenile consumers
*β*	3	Net resource assimilation rate parameter of adult consumers
*μ*_*J*_	0.1	Background mortality rate of juvenile consumers
*μ*_*A*_	0.1	Background mortality rate of adult consumers
*ξ*_*J*_	1	Attack rate parameter of specialist predator on juvenile consumers
*ξ*_*A*_	1	Attack rate parameter of specialist predator on adult consumers
*ɛ*_*J*_	0.1	Conversion efficiency of specialist predator on juvenile consumers
*ɛ*_*A*_	1	Conversion efficiency of specialist predator on adult consumers
*δ*_*J*_	0.1	Background mortality rate of specialist predator on juvenile consumers
*δ*_*A*_	0.1	Background mortality rate of specialist predator on adult consumers
*h*	0.3	Magnitude of the resource competition and predation disadvantage of the double‐handicapped looser in the diamond model

## 3 COMMUNITY CONSEQUENCES OF DENSITY OVERCOMPENSATION

Figure 1 presents results from a very simple and abstract, phenomenological model of which the biological relevance can be disputed. Nonetheless, these results stand example for the response of a wide range of stage‐ and size‐structured consumer–resource models to increases in mortality (de Roos et al., 2007; de Roos & Persson, 2002, 2013). The key ingredients for the stage‐specific density overcompensation (see Glossary) to occur are the juvenile–adult asymmetry in efficiency with which ingested food translates into maturation and reproduction (de Roos et al., 2007), respectively, and the disproportionately large increases in these rates as a result of somatic maintenance costs (de Roos, 2018; de Roos & Persson, 2013). Irrespective of their complexity, density overcompensation in response to increasing mortality is the most important and ubiquitously occurring prediction of stage‐ and size‐structured consumer–resource models. This prediction has been rigorously tested in laboratory populations of the least killifish *Heterandria formosa* (Schröder, Persson, & de Roos, 2009) and has furthermore been shown to occur as well in a variety of other laboratory and field populations (Cameron & Benton, 2004; Nicholson, 1957; Ohlberger et al., 2011; Olson, Green, & Rudstam, 2001; Schröder, van Leeuwen, & Cameron, 2014; Slobodkin & Richman, 1956). In short, stage‐specific density overcompensation in response to (moderate) increases in mortality may very well be the rule in ecological systems.

Even though stage‐specific density overcompensation may seem like a rather esoteric outcome of stage‐ and size‐structured interactions between consumers and their resource, when it occurs its consequences for community structure are substantial and counterintuitive. Consider for example the following extension of the simple consumer–resource model with two stage‐specific predators, *P*_*J*_ and *P*_*A*_, foraging on juvenile and adult consumers, respectively:

(4) $$\begin{array}{l} \frac{\mathrm{dR}}{\mathrm{dt}}=\rho -\mathrm{\alpha RJ}-\mathrm{\alpha RA}\\ \frac{\mathrm{dJ}}{\mathrm{dt}}=\max\left(\mathrm{\beta R}-T,0\right)A-\max\left(\mathrm{\gamma R}-T,0\right)J-\left(\mu +\xi_{J}P_{J}\right)J\\ \frac{dA}{\mathrm{dt}}=\max\left(\mathrm{\gamma R}-T,0\right)J-\left(\mu +\xi_{A}P_{A}\right)A\\ \frac{dP_{J}}{\mathrm{dt}}=\left({ɛ}_{J}\xi_{J}J-\delta_{J}\right)P_{J}\\ \frac{dP_{A}}{\mathrm{dt}}=\left({ɛ}_{A}\xi_{A}A-\delta_{A}\right)P_{A}. \end{array}$$

In this model the two stage‐specific predators *P*_*J*_ and *P*_*A*_ are assumed to forage on juvenile and adult consumers, respectively, following a linear functional response with attack rate *ξ*_*J*_ = 1 and *ξ*_*A*_ = 1, respectively. Their numerical response is proportional to their functional response with proportionality constants *ɛ*_*J*_ = 0.1 and *ɛ*_*A*_ = 1, while they experience default mortality rates equal to *δ*_*J*_ = 0.1 and *δ*_*A*_ = 0.1, respectively. The model parameters for resource and consumers are taken equal to the values discussed in the previous section and also used for Figure 1 with equal background mortality for juvenile and adult consumers, *μ*_*J*_ = *μ*_*A*_ = *μ* = 0.1 (see Table 1).

**FIGURE 2:**
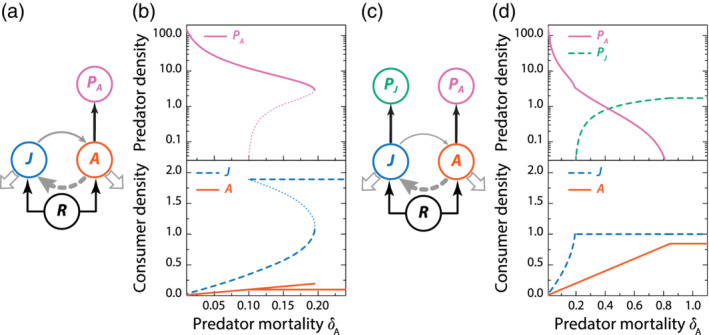
Increased mortality of predators on adult consumers leads to an emergent Allee effect, while the presence of a predator on juvenile consumers facilitates their persistence. (a) Food web module with only predators on adult consumers. (b) As a consequence of the density overcompensation with increasing adult mortality (see Figure 1) a predator foraging exclusively on adult consumers experiences an emergent Allee effect at intermediate mortality (mortality rates between 0.1 and 0.2). In this intermediate range of mortality rates alternative stable equilibrium states occur including a consumer–resource equilibrium without predators (horizontal lines in bottom panel) dominated by high densities of juvenile consumers and a predator–consumer–resource equilibrium with lower and higher densities of juvenile and adult consumers, respectively, than in the consumer–resource equilibrium (folded curves in top and bottom panel; thin dotted line sections represent unstable steady states). (c) Food web module with two predators specializing on juvenile and on adult consumers. (d) In the presence of a predator on juvenile consumers the maximum mortality that a predator on adult consumers can sustain before going extinct is more than four times higher than in the absence of the predator on juveniles (compare maximum mortality rate with predator persistence of 0.2 in panel (b) with the mortality rate > 0.8 at which the predator on adult consumers goes extinct in panel (d)). The predator on juvenile consumers prevents the occurrence of a maturation bottleneck for juvenile consumers and thereby increases the density of adult consumers for the adult‐specific predator to forage on. For model formulation and parameter values see main text. [Color figure can be viewed at wileyonlinelibrary.com]

In the absence of the stage‐specific predator on juveniles *P*_*J*_, the predator on adults *P*_*A*_ is subject to a so‐called emergent Allee effect (de Roos & Persson, 2002; de Roos, Persson, & Thieme, 2003; Van Kooten, de Roos, & Persson, 2005; see Figure 2a,b and Glossary). With increasing mortality *δ*_*A*_ of the stage‐specific predator on adult consumers the density of these predators does not decrease monotonically to reach 0 at a specific threshold value that marks predator extinction. Instead, the curve relating the equilibrium density of the predator to its mortality rate is folded (Figure 2b), which indicates that over a range of predator mortality values (*δ*_*A*_ = 0.1 − 0.2) two alternative stable community equilibrium states are possible, one in which the predator is present, one from which it is absent. The two different equilibrium states differ significantly in the population structure of the consumer population. If the predator is absent the consumer population includes a large density of juveniles and only a small density of adult consumers. If the predator is present, the juvenile consumer density is significantly smaller than its density in absence of the predator. In contrast, the adult consumer density is higher than its density in absence of the predator, despite that the predator exclusively forages on this consumer stage. In the presence of the stage‐specific predator the density of its main prey (adult consumers) is hence increased as a consequence of the density overcompensation that originates from the ontogenetic asymmetry (see Glossary) between juvenile and adult consumers. The emergent Allee effect makes that for mortality levels in the bistable range (*δ*_*A*_ = 0.1 − 0.2) the predator can not establish itself in a consumer–resource equilibrium that it is absent from. Once established, however, the stage‐specific predator enforces a structure on the consumer population with a higher fraction of the consumer population consisting of adults that is to its own benefit and allows the predator to persist in a stable predator–consumer–resource equilibrium. For increasing values of the stage‐specific predator mortality the transition from the stable predator–consumer–resource equilibrium to the stable consumer–resource equilibrium will take place as a catastrophic collapse at a mortality rate around *δ*_*A*_ = 0.2.

Considering the addition of a stage‐specific predator on juvenile consumers to this community module would a priori seem like adding a potential competitor for the stage‐specific predator on adult consumers. Moreover, because the stage‐specific predator on juveniles *P*_*J*_ forages on an earlier life stage of consumer it seemingly has the competitive advantage over the predator on adult consumers. However, nothing can be further from the truth. In the presence of the stage‐specific predator on juvenile consumers the range of mortality levels that the predator on adult consumers can endure before going extinct is four times larger (Figure 2c,d; stage‐specific predator *P*_*A*_ goes extinct around *δ*_*A*_ = 0.8). Also, the equilibrium density of the stage‐specific predator *P*_*A*_ on adult consumers now decreases monotonically with increasing values of its mortality rate *δ*_*A*_, in contrast to the changes in its equilibrium density with mortality in the absence of the stage‐specific predator on juveniles. Because of its foraging on juvenile consumers, the latter keeps the density of juvenile consumers low, such that resource densities are high and hence maturation of juveniles is faster than in the absence of the stage‐specific predator on juveniles. Indirectly, the predator on juveniles thereby increases the density of adult consumers (compare Figure 2b,d), which allows the predator on adults to persist under a wider range of mortality conditions. This indirect positive effect of stage‐specific predators on each other's population growth has been termed emergent facilitation (de Roos, Schellekens, Van Kooten, & Persson, 2008; see Glossary).

The model including two stage‐specific predators discussed here is the simplest model that exhibits the occurrence of the emergent Allee effect and emergent facilitation (see Glossary). These two types of phenomena, which both result from the changes in juvenile–adult population structure with mortality, were first reported in more complex models with more detailed stage‐ or size‐dependent interactions (de Roos et al., 2008; de Roos & Persson, 2002). Emergent Allee effects and facilitation have moreover been shown to be robust to adding more model detail, for example, by accounting for a complete size‐distribution of the predator population with more complex foraging interactions depending on the body size of both predators and consumers (de Roos & Persson, 2013). Furthermore, an emergent Allee effect has been shown to occur in an empirical system involving brown trout and Arctic char (Persson, Amundsen, et al., 2007; Persson et al., 2013), while the occurrence of emergent facilitation has been experimentally supported as well (Huss & Nilsson, 2011). Lastly, whereas the facilitation is in the example case discussed here always unilateral, mutual facilitation between stage‐specific predators can also occur when juvenile and adult consumers exploit different resource (de Roos & Persson, 2013).

The effect of changing population structure on communities involving competing species has been considered to a much lesser extent than its effect in multi‐trophic communities. Schellekens, de Roos, and Persson (2010) showed in a community involving two consumer species and two resources that the two competitors can coexist if they differ in their stage‐specific preferences for the two resources. Similarly, Schellekens and Van Kooten (2012) have considered how stage‐specific differences in the diets of two adult intraguild predators that feed on each others juvenile stages in addition to sharing a basal resource promote coexistence of these competitors. In these results, however, the role of population structure is not as prominent as its role in the emergent Allee effect and facilitation, given that, for example, two competitors can coexist while competing for two basal resources even when population structure effects are not considered.

However, also in communities involving different competitors accounting for population structure may lead to theoretical predictions about community structure that are counterintuitive and contradict established theory based on models without population structure. Consider for example the diamond web, in which two consumers compete for a shared resource and are preyed upon by a shared predator (Grover, 1995; Grover & Holt, 1998; Holt, Grover, & Tilman, 1994; Leibold, 1996). In general, classic theory based on models without population structure predicts that the superior resource competitor will prevail at low productivity of the basal resource, when predation mortality hardly plays any role, whereas the competitor that is less vulnerable to predation will outcompete its opponent at high productivity when the shared predator is also abundant and imposes significant mortality (Grover & Holt, 1998). This theoretical prediction clearly aligns with our intuition that competitors that most efficiently exploit the resource will dominate when competition is important whereas competitors that are best protected dominate when predation determines community composition. And, a Darwinian demon‐like species that excels at both resource competition and protection against predators will always outcompete a “double‐handicapped looser” that is inferior to it in both aspects. This obvious expectation is however overturned if population structure of competitors is considered.

Take the following model for competition for a shared resource *R* while exposed to a shared predator *P* between juveniles and adults of a species *C* without any juvenile–adult asymmetry, the densities of which are indicated with *J*_*C*_ and *A*_*C*_, respectively, and juveniles and adults of a species *D*, in which asymmetry between juveniles with density *J*_*D*_ and adults with density *A*_*D*_ does occur:

(5) $$\begin{array}{l} \frac{\mathrm{dR}}{\mathrm{dt}}=\rho -\mathrm{\alpha R}\left(J_{C}+A_{C}+J_{D}+A_{D}\right)\\ \frac{dJ_{C}}{\mathrm{dt}}=\max\left(\beta^{\prime }R-T,0\right)A_{C}-\max\left(\beta^{\prime }R-T,0\right)J_{C}-\left(\mu +P\right)J_{C}\\ \frac{dA_{C}}{\mathrm{dt}}=\max\left(\beta^{\prime }R-T,0\right)J_{C}-\left(\mu +P\right)A_{C}\\ \frac{dJ_{D}}{\mathrm{dt}}=\max\left(\mathrm{\beta R}-T,0\right)A_{D}-\max\left(\mathrm{\gamma R}-T,0\right)J_{D}-\left(\mu +\left(1+h\right)P\right)J_{D}\\ \frac{dA_{D}}{\mathrm{dt}}=\max\left(\mathrm{\gamma R}-T,0\right)J_{D}-\left(\mu +\left(1+h\right)P\right)A_{D}\\ \frac{\mathrm{dP}}{\mathrm{dt}}=\left(J_{C}+A_{C}+\left(1+h\right)\left(J_{D}+A_{D}\right)\right)P-\mathrm{\delta P}. \end{array}$$

The productivity of the resource in this model is assumed constant as before at *ρ* = 20. The two competing species are modeled as much as possible the same with linear functional responses and identical attack rates *α* = 10, identical background mortality rates for both juveniles and adults *μ*_*J*_ = *μ*_*A*_ = *μ* = 0.1 and identical values for the threshold parameter *T* = 1. The shared predator density is expressed in scaled units such that its attack rate on species *C* equals 1 and its mortality rate equals *δ* = 1. The main differences between the two competitors are that predators forage on species *D* at a rate 1 + *h*, in which the parameter *h* = 0.3 represents the predation handicap of species *D*. Juvenile maturation of species *D* is more limited by resource than its reproduction and halts altogether at resource levels below *T*/*γ* and *T*/*β*, respectively, with *γ* = 1 and *β* = 3 (see Table 1). Above these threshold resource densities juvenile maturation and adult fecundity increase linearly with resource with the same scaling constants with *γ* = 1 and *β* = 3, respectively. In contrast, juvenile maturation and adult fecundity are assumed to be equally limited by resource for species *C*, coming both to a halt for resource levels below *T*/*β*^′^ and increasing linearly with resource above this threshold with proportionality constant *β*^′^. The value of the constant *β*^′^ is now chosen in such a way that the resource density $R_{D}^{*}$ in a consumer–resource equilibrium with only species *D* is (1 + *h*) times larger than the resource density $R_{C}^{*}$ in a consumer–resource equilibrium with only species *C*. The single parameter *h* thus measures the competitive disadvantage in terms of R^*^ values of species *D* relative to species *C* and its predation disadvantage in terms of the predation mortality it experiences relative to species *C*. In particular, from the equations above the following expression can be derived for the resource density in a consumer–resource equilibrium with only species *D*:

$$R_{D}^{*}=\frac{\left(\beta +\gamma \right)T+\gamma \mu +\sqrt{{\left(\left(\beta +\gamma \right)T+\gamma \mu \right)}^{2}-4\beta \gamma \left(T^{2}+\mathrm{T\mu}-\mu^{2}\right)}}{2\beta \gamma }.$$

Similarly, the resource density in a consumer–resource equilibrium with only species *C* can be derived to equal:

$$R_{C}^{*}=\frac{T+\mu \left(\frac{1}{2}+\frac{1}{2}\sqrt{5}\right)}{\beta^{\prime }\left(h\right)}.$$

For $R_{D}^{*}$ to equal $\left(1+h\right)R_{C}^{*}$ the scaling parameter *β*^′^(*h*) therefore has to be chosen equal to:

$$\beta^{\prime }\left(h\right)=\frac{2\beta \gamma \left(T+\mu \left(\frac{1}{2}+\frac{1}{2}\sqrt{5}\right)\right)}{\left(\beta +\gamma \right)T+\gamma \mu +\sqrt{{\left(\left(\beta +\gamma \right)T+\gamma \mu \right)}^{2}-4\beta \gamma \left(T^{2}+\mathrm{T\mu}-\mu^{2}\right)}}\left(1+h\right).$$

**FIGURE 3:**
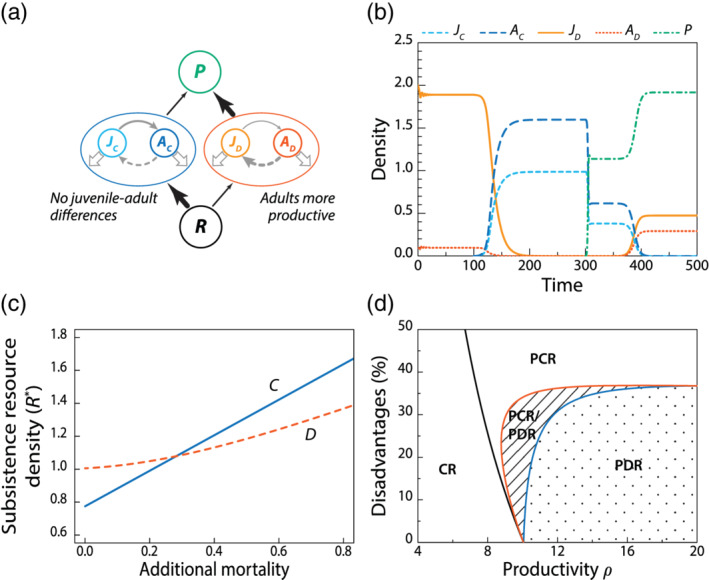
Juvenile–adult asymmetry allows a more predator‐sensitive and inferior resource competitor to exclude its opponent. (a) Diamond module with a dominant resource competitor *C* without any differences between juveniles and adults (*β* = *γ* = *β*^′^ = 1.5) and a consumer *D* with juvenile–adult asymmetry (*β* = 3.0, *γ* = 1.0) exposed to a shared predator that forages at a 30% higher rate on consumer *D*, but does not discriminate between juveniles and adults of either species. (b) A consumer–resource system with only consumer species *D* and dominated by juveniles is invaded at *t* = 100 by consumer *C*, which quickly outcompetes *D* because the latter requires a 30% higher resource density for persistence ($R_{C}^{*}=0.77,R_{D}^{*}=1.0$). Invasion of the shared predator that forages indiscriminately on juveniles and adults allows consumer *D* to recover and eventually even exclude its competitor *C*, because predation mortality increases the fraction of adult consumers *D*_*A*_ that are more effectively using ingested food for reproduction than juvenile consumers *D*_*J*_. (c) Resource density required for persistence (R^*^) of the competitors *C* and *D* at different levels of (predation) mortality in addition to their background mortality, showing that at higher mortality species *D* can persist at lower resource densities than species *C* despite its competitive handicap at low mortality. (d) Parameter combinations with (CR) a unique consumer–resource equilibrium with only consumer *C* (PCR) a unique predator–consumer–resource equilibrium with only consumer *C* (PDR) a unique predator–consumer–resource equilibrium with only consumer *D* and (PCR/PDR) alternative stable predator–consumer–resource equilibria including either consumer *C* or consumer *D*. Coexistence of consumer species *C* and *D* is not observed for any of the parameter combinations analyzed. The disadvantage represented on the *y*‐axis indicates how much higher the R^*^‐value as well as the predation mortality rate is of consumer *D* compared to consumer *C*. For model formulation and parameter values see main text. [Color figure can be viewed at wileyonlinelibrary.com]

Figure 3b shows the results of a simulation of the diamond model (5) for a 30% competitive and predation handicap of species *D* (*h* = 0.3). Starting in an equilibrium state with only species *D* and the basal resource, invasion of species *C* quickly leads to its establishment and the disappearance of species *D* as a result of its 30% higher resource requirement (R^*^). Subsequent invasion of the predator initially leads to a reduction in density of species *C* by some 60%, which at the same time allows species *D* to recover from its low density. After recovering, species *D* even outcompetes its opponent, which ultimately goes extinct. The higher equilibrium density of predators that species *D* supports contributes to the extinction of *C*. Despite substantial disadvantages of species *D* in terms of both resource competition and vulnerability to predation, this “double‐handicapped looser” drives its opponent to extinction as a result of the difference in juvenile–adult asymmetry between the species. Whereas in the absence of predation the population of species *D* consists for 95% of juveniles, in the presence of the predator it consists of some 60% juvenile and 40% adult individuals. Since the adult individuals use ingested resource more effectively for population growth, the change in population structure brought about by the predator increases the resilience of the population of species *D*. In contrast, the population of species *C* is always characterized by the same ratio between juvenile and adult consumers as a result of the symmetry in maturation and fecundity. This change in population structure and its consequence that resources are used more effectively for population growth makes that species *D* can persist at lower equilibrium resource densities than species *C* at higher mortality rates (Figure 3c), despite its competitive disadvantage at low mortality.

Figure 3d shows for different values of the system productivity *ρ* and different values of the magnitude of the double handicap *h* the possible community equilibrium states predicted by model (5). For most of the values of system productivity that allow predators to establish itself in an equilibrium state of species *C* and the basal resource, the presence of the predator allows species *D* to establish itself and drive species *C* to extinction, unless the R^*^‐value of species *D* in the absence of predation and the predation mortality it experiences are more than 35% higher than the corresponding values for species *C*. In a small range of parameters alternative stable community states can occur with either species *C* or species *D* coexisting with the predator and the basal resource, but for substantial ranges of parameters the double‐handicapped looser outcompetes the superior species *C* as a result of its juvenile–adult asymmetry in maturation and fecundity. Coexistence between the two consumer species is not observed for any of the parameter combinations investigated.

## 4 DISCUSSION

In this paper I have first of all summarized the necessary requirements for density overcompensation to occur in consumer–resource systems (de Roos, 2018). Second, using the simplest model that allows for density overcompensation to occur I have highlighted some of its consequences for the possible community equilibria in multi‐trophic systems. Last, I have presented new results on communities, in which two species compete for a shared resource and are exposed to a shared predator.

Density overcompensation was shown to result from an increase in efficiency with which ingested resource translates into consumer population growth at higher levels of consumer mortality. This increase in efficiency emerges naturally if different life history stages are limited to a different extent by food availability and maturation, growth and reproduction come to a halt at low resource levels because all ingested food is needed to cover somatic maintenance costs. Somatic maintenance costs are a fact of life, while numerous studies have also documented asymmetric competition for resource between different life history stages (Briggs, Sait, Begon, Thompson, & Godfray, 2000; Byström & Andersson, 2005; Cameron, Wearing, Rohani, & Sait, 2007; Hamrin & Persson, 1986; Persson, 1988; Potter, King, Travis, & Bassar, 2018). Density overcompensation can therefore be expected to occur in many systems and under a wide range of conditions. In this paper I have focused on the increase in numerical abundance of a particular life history stage with an increase in mortality, whereas previous studies have focused more on the increase in stage‐specific biomass (de Roos et al., 2007; de Roos & Persson, 2002, 2013). The necessary requirements for overcompensation in either numerical abundance or biomass are however the same as both rely on asymmetry between juveniles and adults (de Roos et al., 2007, 2013) and an explicit consideration of maintenance requirements (de Roos & Persson, 2013, p. 490). By and large the density or biomass overcompensation emerges because an increase in mortality reduces the losses to somatic maintenance and channels a larger fraction of ingested food into life history processes like maturation and reproduction that effectively contribute to population growth rather than population stasis.

Overcompensation in the numerical abundance or biomass density of a particular life history stage is especially remarkable when it occurs in response to increases in mortality imposed on the same stage. In the simple model discussed in this paper, this can only occur in the adult stage, but in size‐based models that in addition to maturation and reproduction also account for somatic growth of immature individuals it can occur in juvenile life history stages as well (de Roos et al., 2007; de Roos & Persson, 2002, 2013). Moreover, its occurrence in juvenile life stages has been rigorously tested in experiments with self‐sustaining fish populations (Schröder et al., 2009). More generally, overcompensation has been argued to occur in a variety of systems (Schröder et al., 2014) even though it is not directly a prominent topic of research. Stage‐specific overcompensation in response to increasing mortality, however, is crucial for understanding the effect of life history and intraspecific competition on community structure, as it is the basic ingredient for the occurrence of the emergent Allee effect (de Roos & Persson, 2002; Persson, Amundsen, et al., 2007) as well as emergent facilitation (de Roos et al., 2008).

This paper has clearly focused on counterintuitive results that are qualitatively different from expectations based on models without population structure. Classic, unstructured models of food chains, for example, predict that the number of trophic levels increases with system productivity and decrease with predator exploitation rate (Oksanen, Fretwell, Arruda, & Niemela, 1981), but that otherwise equilibrium densities of all species vary smoothly with changes in productivity or predator exploitation. In contrast, as exemplified by the emergent Allee effect stage‐ or size‐structured models predict the occurrence of alternative stable equilibria under the same conditions of productivity and exploitation with catastrophic shift occurring between these states when varying these environmental factors. Similarly, both intuitively and theoretically it would be considered impossible that a species that is outcompeted under conditions of strong resource competition and also suffers more from predation is the ultimately dominant consumer in a diamond food web. That stage‐specific density overcompensation due to juvenile–adult asymmetries makes this a potential outcome of community dynamics underlines the importance of considering population structure and not just abundances when studying ecological communities. In addition to qualitative differences, the consideration of population structure can also have effects that are more quantitative in nature. Miller and Rudolf (2011) review a range of such effects plus the questions that emerge from them and call for more analysis of the role of population structure in community ecology, similar to the argumentation in this paper.

The insights about the effects of life history on community dynamics and structure that are derived from the stage‐structured models also have repercussions for experimental and empirical research. In a general sense these theoretical insights stress the importance of adopting a life history perspective. Too often in experimental or empirical research the focus is on only part of the life cycle of the species that is studied. For example, in many studies the fitness of individuals is equated with the number of offspring produced. Such an assumption may or may not be appropriate depending on what process in the individual life history limits population dynamics most, juvenile growth and maturation or adult reproduction. Similarly, from the perspective of a single individual mortality is always a negative effect, but considering mortality in the context of the entire life history and the interactions between individuals in different stages of their life the view changes and mortality can even be considered to have a positive influence (Schröder et al., 2014). Even though it is most often the subject of study an individual organism is much more than its adult phenotype with its associated traits. In fact, the individual can best be identified with its entire life history and this life history is not just the product of its genetic makeup but is shaped by its ecological context as well. Such a life history perspective is necessary if we aim to better understand the dynamics and structure of ecological systems.

## 5 FUTURE PERSPECTIVES

From a theoretical point of view the results summarized in this paper suggest the need of a shift in perspective: Instead of adopting unstructured population models as a paradigm framework and asking the question how accounting for life history or stage structure changes the predictions of these unstructured models, stage‐structured models that include key aspects of the individual life history should be adopted as models of choice. I argue that maintenance costs and juvenile–adult differences are two of these key aspects. The important question to ask is then to what extent the predictions derived from these structured models are robust against ignoring individual life history. This shifts the focus from questioning the relevance of stage‐structured models to questioning the relevance of unstructured models for understanding dynamics of ecological communities. Theory development has a long history in ecology and has generated a considerable body of theory that by and large is all based on unstructured population models. Up to now the analysis of structured models for community interactions has focused on only a limited part of this ecological theory with already a substantial number of new or changed insights. Below I discuss a number of potential future research directions to expand on that.

One important direction of future research concerns different types of interactions. Current analyses of structured models for community interactions have primarily focused on predator–prey interactions. Some preliminary results documenting potential effects of stage‐structure on competitive interactions are presented in this paper, but stage‐dependent competition between different species is definitely a subject that needs further investigation. Interactions such as mutualism, symbiosis, or parasitism are often stage specific as well but have only been explored to a limited extent with stage‐structured models (Ke & Nakazawa, 2018; Nakazawa, 2020). Murdoch et al. (2003) have summarized a substantial body of theory on the interaction between stage‐structured hosts and their parasitoids, but their results provide little insight about the consequences of host–parasite interactions for community structure and energetics. Given that diseases and parasites increase the energetic costs of their host and maintenance costs play such an important role in stage‐structured interactions, stage‐structured models of host–parasite interactions are therefore another promising topic for future research, especially in the context of larger ecological communities. For example, parasites exploit the energy assimilated by their hosts and thereby reduce host somatic growth and reproduction (Civitello, Fatima, Johnson, Nisbet, & Rohr, 2018; Hall, Simonis, Nisbet, Tessier, & Cáceres, 2009). By reducing host density and hence increasing resource availability for surviving hosts, predators might actually promote the spread of parasites through the host population rather than suppress parasite prevalence.

A second direction of future theoretical research pertains to changes in the nature rather than the strength of ecological interactions that individuals engage in during their life history. In this paper I only considered juvenile–adult asymmetry due to intrinsic differences in the use of ingested resources. Such asymmetry can, however, also result from shifts in diet during ontogeny such that juveniles and adults feed on different resources with different productivities. Such ontogenetic niche or diet shifts are part of the life history of many species (Werner & Gilliam, 1984; Wilbur, 1980). Schreiber and Rudolf (2008) showed that ontogenetic diet shifts allow for the occurrence of alternative stable community states that differ in that they are either dominated by juveniles or by adults. At low productivities of the resource that juvenile consumers forage on, maturation is the bottleneck in the consumer life history, whereas reproduction is the most rate‐limiting life history process if the productivity of the resource that adults feed on is low. Gradual changes in either juvenile or adult resource supply can in this case lead to abrupt shifts between these alternative community states.

The type of interaction between two particular species may also change during life history of the individuals involved. The interaction between many piscivorous fish species and their fish prey often changes from a competitive relation early in life to a predation interaction later on (Wilbur, 1988). Comparable changes in the type of interaction occur between, for example, butterflies and their host plants, when caterpillars feed on the host plant, but the butterflies are essential for plant pollination. The implications of such ontogenetic niche shifts in a wider community context have recently been discussed by Nakazawa (2010, 2011, 2015), who also point out that currently an ontogenetic perspective in community ecology is still far from complete. Given the complexity of life cycles that can be observed in nature the impact of ontogenetic niche shifts in the widest sense on community structure is a field of study that is theoretically explored to a very limited extent.

Lastly, to what extent stage‐structure influences the diversity, complexity and stability of large ecological communities is an important topic of future study. The consequences of ontogeny and population structure for community structure have been studied in small community modules only, involving a few species that act as resources, consumer and predators. Even though the study of community responses due to stage‐structured interactions is gaining momentum (e.g., Lindmark, Huss, Ohlberger, & Gårdmark, 2017; Lindmark, Ohlberger, Huss, & Gårdmark, 2019; Pessarrodona, Boada, Pagès, Arthur, & Alcoverro, 2019) the real challenge is whether and to what extent effects of ontogeny and population structure will occur in large networks of interacting species. Despite that the analysis of empirical food webs has revealed that interactions between species are strongly dependent on body size (Nakazawa, Ushio, & Kondoh, 2011; Woodward, Ebenman, et al., 2005; Woodward & Hildrew, 2002; Woodward, Speirs, & Hildrew, 2005) the currently established approach is to model the interactions between large numbers of species with populations as basic unit of representation and the network of interspecific interactions characterized by the community matrix (Allesina & Tang, 2015; Barabás, Michalska‐Smith, & Allesina, 2017). The challenge for future research is to reveal to what extent the counterintuitive consequences of ontogenetic asymmetry also impact or change the currently established predictions of food web ecology.

## GLOSSARY

**Biomass overcompensation:** An increase in the equilibrium biomass density of a consumer in response to increasing mortality. Biomass overcompensation was introduced by de Roos et al. (2007) and shown to occur in a stage‐structured biomass model for a consumer–resource interaction, accounting for both juvenile and adult consumers. The increase in equilibrium consumer biomass can be limited to a single consumer stage or can occur in total consumer biomass (de Roos & Persson, 2013). Biomass overcompensation differs from the Hydra effect not only because it involves biomass rather than numerical density but also because of its mechanistic basis. Biomass overcompensation occurs irrespective of whether mortality of the entire population or of a particular life history stage increases, because with increasing consumer mortality relatively more of the resources ingested by consumers is spent effectively on population growth through somatic growth, maturation and reproduction rather than on metabolic maintenance requirements.

**Density overcompensation:** An increase in the stage‐specific equilibrium density of a consumer in response to increasing mortality. Density overcompensation was shown to occur by de Roos (2018) in a stage‐structured model for a consumer–resource interaction, accounting for both juvenile and adult consumers. It is similar to biomass overcompensation but occurs in stage‐structured models in terms of numerical densities of juvenile and adult consumers, which hence do not account for somatic growth of individuals. Density overcompensation differs from the Hydra effect in its mechanistic basis, as it occurs because with increasing consumer mortality relatively more of the resources ingested by consumers is spent effectively on population growth through maturation and reproduction rather than on metabolic maintenance requirements.

**Double‐handicapped looser:** A consumer species that is competitively inferior to its competitor and is hence outcompeted in absence of predators and also experiences higher mortality then its competitor when a shared predator is present. Differences in ontogenetic asymmetry allow double‐handicapped losers to nonetheless oust their opponents in the presence of predators, while competing for the same resource.

**Emergent Allee effect:** An Allee effect of a predator on a structured prey population that emerges from the change in stage‐ or size‐structure of the prey with increasing mortality. The predator experiences positive density dependence at low density (an Allee effect) because the density of its preferred prey stage increases through density or biomass overcompensation despite a decreasing overall prey density.

**Emergent facilitation:** The (mutual) facilitation between two stage‐ or size‐selective predators of the same structured prey population, which emerges from the changes in prey stage‐ or size‐structure that the predators induce through the occurrence of density or biomass overcompensation in the prey population.

**Hydra effect:** An increase in the equilibrium or time‐averaged density of a consumer population with increasing mortality. The Hydra effect was introduced by Abrams and Matsuda (2005) as a phenomenon occurring in consumer–resource models without any population structure. The Hydra effect differs from density or biomass overcompensation in its mechanistic basis because it results from an increase in resource productivity when resource density increases due to the increased mortality of its consumers. A combination of logistic resource growth and saturating resource ingestion rate of the consumer are hence essential ingredients for hydra effects which only occur when unstructured consumer populations exhibit cyclic dynamics.

**Juvenile–adult asymmetry:** See ontogenetic asymmetry.

**Ontogenetic asymmetry:** Differences between individuals of different body sizes or life‐history stages in ecologically relevant vital rates, such as resource ingestion rate, biomass maintenance rate, or resource and habitat use. Ontogenetic asymmetry translates into differences in individual competitiveness and therefore determines which life stage constitutes a bottleneck to population growth.
